# Acupuncture for allergic rhinitis: protocol for a systematic review and network meta-analysis

**DOI:** 10.3389/falgy.2024.1499406

**Published:** 2024-12-09

**Authors:** YanNi Chen, ChenFei Song, JiaQin Wang, XinMin Han

**Affiliations:** ^1^ First Clinical Medical College, Nanjing University of Chinese Medicine, Nanjing, China; ^2^ Department of Traditional Chinese Pediatrics, Shanghai Municipal Hospital of Traditional Chinese Medicine, Shanghai, China; ^3^ TCM Department, Shanghai Baoshan District Youyi Street Community Health Service Center, Shanghai, China; ^4^ Department of Traditional Chinese Pediatrics, Jiangsu Province Hospital of Chinese Medicine, Nanjing, China

**Keywords:** allergic rhinitis, acupuncture, network meta-analysis, randomised control trial, protocol

## Abstract

**Introduction:**

Allergic rhinitis (AR) is a widespread inflammatory disorder of the nasal mucosa affecting millions globally. The increasing prevalence of AR underscores the need for effective treatment modalities. Acupuncture has been identified as a potential non-pharmacological intervention for AR due to its effects on autonomic nerve functions and neuroendocrine and immune networks. However, a comprehensive evaluation of different acupuncture techniques through high-quality, evidence-based research is still needed.

**Methods and analysis:**

Randomised controlled trials of different acupuncture-related therapies for postmenopausal osteoporosis will be searched in the following databases from 1 January 2002 to 31 December 2022, including PubMed, Embase, Cochrane Library, Web of Science, China National Knowledge Infrastructure, VIP Database, Wanfang Database and China Biomedical Literature Database. Overall, clinical efficacy rate, bone mineral density and a Visual Analogue Scale score are used as the primary outcome indicators. In addition, the secondary outcome indicator is adverse reactions. Meanwhile, Stata (V.14.0) and RevMan (V.5.4) will be used to conduct the network meta-analysis. If the data are permissible and feasible, we will also perform meta-regression and subgroup analyses to address the underlying causes of data inconsistency and heterogeneity in the statistical analyses. To improve the credibility of this network meta-analysis, we will evaluate the quality of evidence in this research according to the GRADE assessment.

**Prospero Registration Number**: identifier (CRD 42024579713).

## 1 Introduction

Allergic rhinitis (AR) is a prevalent disease of the upper respiratory tract that impacts a substantial portion of the global population (1). It is estimated that over 400 million individuals globally suffer from AR, which manifests with symptoms including sneezing, runny nose, and nasal congestion (2, 3). The pathophysiology of AR involves an immunological reaction in the nasal mucosa, which is triggered by inhaled allergens and mediated by Immunoglobulin E (IgE). This IgE-mediated response is characteristic of the body's hypersensitivity to environmental allergens (4, 5).In the United States, the impact of AR is significant, resulting in approximately 3.5 million lost workdays and 2 million missed school days annually. The economic implications are considerable, extending beyond direct healthcare costs to include reduced productivity and increased absenteeism (6, 7).

AR is managed using diverse strategies, which include the avoidance or elimination of allergens, non-pharmacological therapies, and pharmacological interventions. Common pharmacological treatments encompass antihistamines, leukotriene receptor antagonists, and topical corticosteroids. Additionally, innovative treatments such as sublingual immunotherapy (8), probiotic interventions (9), and herbal medicines (10) are gaining traction due to their promising results in AR management. Clinical guidelines further endorse non-pharmacological methods as valuable clinical interventions for AR due to their safety, cost-effectiveness, and minimal impact on lifestyle (11). Saline nasal irrigation and acupuncture are notable examples. Saline irrigation helps alleviate nasal congestion by removing allergens and irritants from the nasal passages. Meanwhile, acupuncture has been recognized in the 2015 American guidelines for the management of AR as an optional treatment, which reflects its increasing acceptance and reported efficacy in some patient cohorts (12). In 2018, the Chinese Allergy Society's Guidelines for the Diagnosis and Treatment of AR included acupuncture as a treatment for AR (13). At present, acupuncture has been widely used in China and the United States to treat AR diseases (14–16). For patients with AR, it is of great significance to find an effective, simple and safe way to treat AR.

Acupuncture, recognized as a complementary and alternative medicinal practice, has gained widespread acceptance and application globally (17). Numerous studies have demonstrated that acupuncture can effectively alleviate symptoms of AR such as nasal congestion, rhinorrhea, and sneezing, with lasting and stable effects (18). It has been identified that dysfunction of the autonomic nervous system plays a significant role in the exacerbation of AR (19). Acupuncture has proven beneficial in alleviating conditions associated with autonomic nervous dysfunction, and this may extend to its use in AR treatment as well (20). Moreover, acupuncture may exert its anti-inflammatory effects through various neuroendocrine and immune network pathways, thereby preventing or reducing the occurrence of AR. Further research has suggested that acupuncture's regulatory effects on nasal mucosal immune function are potentially connected to decreased levels of neuropeptide Substance P and specific IgE, thereby mitigating the allergic reactions within the nasal cavity (21). Despite the popularity of acupuncture in treating AR, significant gaps remain in the literature regarding the comparative efficacy of different acupuncture techniques. Currently, no consensus exists on the most effective acupuncture method due to the diversity and variability in techniques applied across clinical studies. This lack of definitive guidance creates clinical uncertainty, making it challenging for healthcare providers to recommend the most effective acupuncture treatment plan for AR patients. Systematic review and network meta-analysis (NMA) seek to address these crucial gaps by empirically exploring and comparing the efficacy of various acupuncture therapies.

NMA is a method that allows for the simultaneous comparison of multiple treatments within a single meta-analysis (22), thus identifying the potentially optimal treatment choice. In this study, we aim to develop a protocol for a NMA that will assess the efficacy and safety of various acupuncture therapies in treating AR. This protocol will outline the methods for systematically evaluating existing clinical trial data to identify the most effective acupuncture techniques.

## 2 Methods and analysis

This NMA is conducted rigorously following the PRISMA 2020 guidelines (23), as outlined in the Supplementary File. Furthermore,to enhance transparency and facilitate verification of our methods, the study is registered with PROSPERO, bearing the registration number CRD 42024579713.

### 2.1 Objectives

Primary Objective: to determine the efficacy of different acupuncture techniques in reducing the Total Nasal Symptom Score (TNSS) for patients with AR.

Secondary Objective: To assess the overall impact of acupuncture on clinical improvement, ocular discomfort, immunological markers, recurrence rates, and adverse events associated with treatment.

### 2.2 Inclusion and exclusion criteria

#### 2.2.1 Population

The eligible population for this NMA will include patients diagnosed with AR. There will be no restrictions based on gender, age, or duration of disease. Furthermore, it is imperative that the original studies report the diagnostic criteria for AR (e.g., criteria outlined as specified in clinical guidelines, or the inclusion criteria for study participants). It is crucial for the inclusion of studies that they provide a clear description of the diagnostic criteria used for AR. These criteria must align with those outlined in recognized clinical guidelines or standard diagnostic frameworks to ensure consistency across studies.

#### 2.2.2 Interventions

Acupuncture is defined as any acupoint-based therapy, irrespective of the needling or stimulation technique. Eligible interventions may include single or combined acupuncture techniques, or acupuncture with other therapies identical to the control group. Non-acupoint stimulating interventions will be excluded.

#### 2.2.3 Comparisons

Guideline-recommended therapies for AR (e.g., Western medication, lifestyle interventions, supplements), combined therapies, different acupuncture methods, and inactive interventions like sham acupuncture, waiting lists, and placebos.

#### 2.2.4 Outcomes

(1) Primary outcome: The primary outcome measure, TNSS, is quantified on a scale from 0 to 12, where a lower score denotes fewer symptoms. This score encapsulates four primary nasal symptoms, each evaluated on a scale from 0, indicating no symptoms, to 3, indicating symptoms severe enough to disrupt daily activities or sleep. (2)Secondary Outcomes: ① Efficacy: Assessed through clinical improvement and symptom relief. ② Total Ocular Symptom Score (TOSS): the sum of patient-reported scores for individual ocular symptoms, quantifying the overall severity of ocular discomfort. ③ Total Symptom Score (TSS): a composite score that combines various types of rhinitis symptoms, including nasal, ocular, and/or palatal symptoms. ④ Immunological Markers: Serum levels of IgE and cytokines IL-4, IL-10, and IL-33. ⑤ Recurrent Rate: Frequency of symptom recurrence post-treatment. ⑥ Adverse Events: Documentation and analysis of any negative effects or complications arising from the treatment.

#### 2.2.5 Study Designs

Only randomized controlled trials (RCTs) reported in English or Chinese will be included, without geographical limitations. Exclusions apply to non-RCTs, animal experimental studies, case studies, expert opinions, and other types of research.

### 2.3 Databases and search strategies

To ensure a comprehensive and reliable search for studies on acupuncture treatments for AR, we will conduct a detailed search spanning multiple databases and clinical trial registries up to September 2024. This includes eight major databases: Pubmed, Web of Science, Embase, Scopus (for English-language literature), and China National Knowledge Infrastructure, Wanfang Database, Chinese Biomedical Literature Database, VIP Database for Chinese Technical Periodicals (for Chinese-language literature).

Additionally, to uncover unpublished data and ongoing trials, we will search five key clinical trial registries: the National Institute of Health Clinical Registry, the Chinese Clinical Trial Registry, the Australian New Zealand Clinical Trials Registry, the International Clinical Trial Registration Platform (ICTRP), and the ISRCTN registry. The Cochrane Central Register of Controlled Trials (CENTRAL) will also be searched for both published and unpublished trials.

To ensure a comprehensive search in Pubmed for studies on acupuncture treatments for AR, we will conduct an advanced search using both medical subject headings (MeSH) terms and a combination of keywords and free text terms optimized for this database. The search strategies are exemplified using PubMed, as detailed in Table 1. We will adapt these strategies to accommodate the unique constraints and features of each database utilized in the study, including the incorporation of both English and Chinese terms when searching in Chinese databases like CNKI and Wanfang. For example, in CNKI, “acupuncture” will be searched as “针灸” and “allergic rhinitis” as “过敏性鼻炎”.

**Table 1:** Search strategy for PubMed database.

No.	Search terms
#1	MeSH terms: “rhinitis, allergic”
#2	Title/Abstract: “allergic rhinitides” OR “rhinitides, allergic” OR “allergic rhinitis”
#3	#1 OR #2
#4	MeSH terms: “acupuncture” OR “acupuncture therapy” OR “acupuncture, ear” OR “acupuncture points” OR “acupressure”
#5	Title/Abstract: “acupuncture” OR “manual acupuncture” OR “electroacupuncture” OR “body acupuncture” OR “auricular acupuncture” OR “electro-acupuncture” OR “auricular acupressure” OR “warm needling” OR “warm acupuncture” OR “fire acupuncture” OR “acupoint” OR “acupressure” OR “needle*” OR “needling”
#6	#4 OR #5
#7	Publication Type: “randomized controlled trial”
#8	MeSH terms: “randomized controlled trials as topic”
#9	Title/abstract: “randomized” OR “randomly” OR “RCT” OR “trial”
#10	#7 OR #8 OR #9
#11	#3 AND #6 AND #10

### 2.4 Selection of studies

All articles from databases will be organized using EndNote (version X9, Clarivate, USA), with duplicates removed. Two reviewers (YNC and CFS) will independently screen these articles by reviewing titles, abstracts, and full texts against the inclusion criteria. For unclear studies, authors will be contacted for clarification. To resolve discrepancies during the selection phase, the two primary reviewers will first attempt to reach a consensus through discussion. If disagreements remain, a third reviewer (XMH) will make the final decision to ensure impartiality and maintain process integrity.

**Figure 1:**
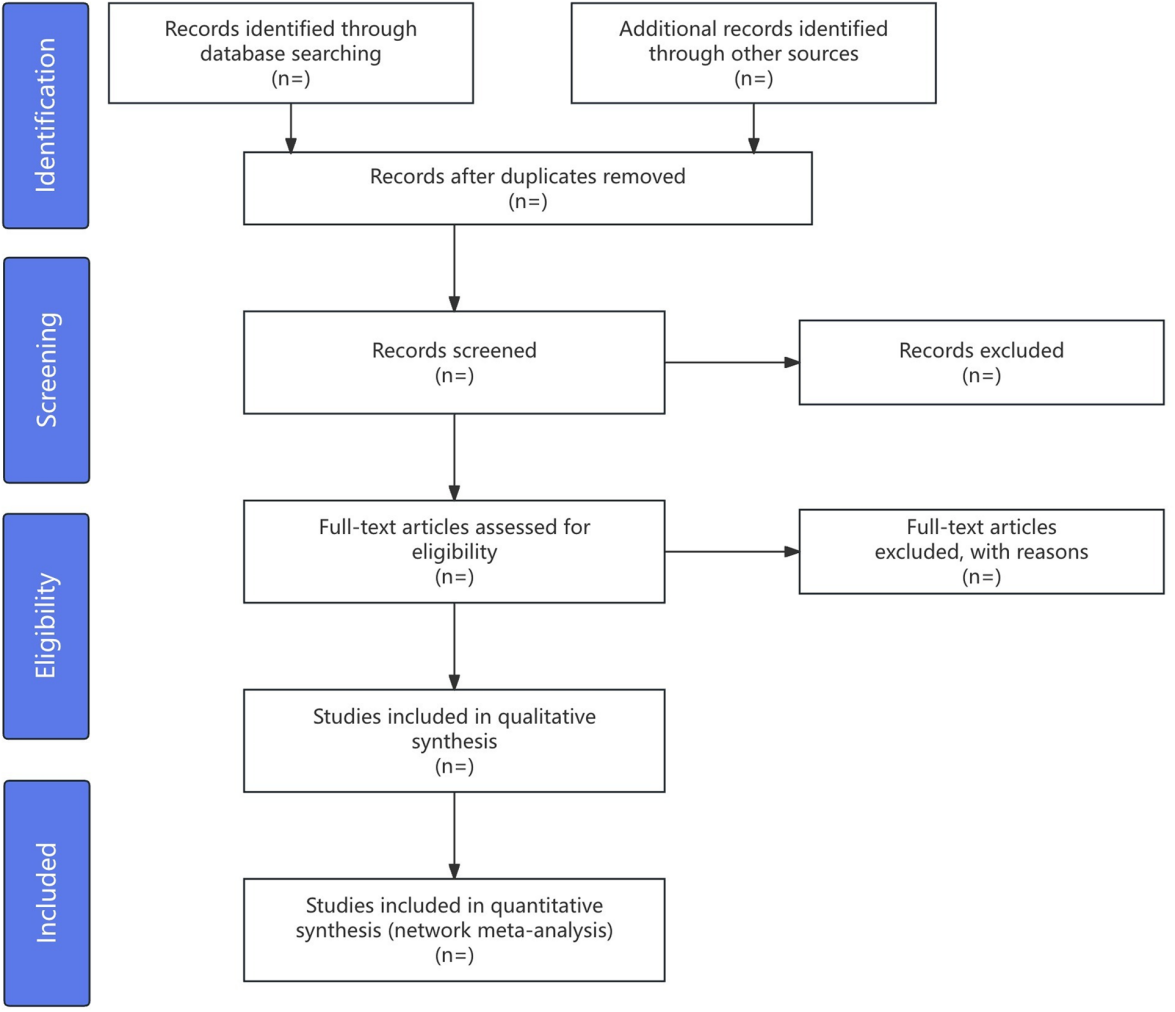
The PRISMA flow diagram of the study selection process.

Data extraction will be conducted using a standardized Excel form by reviewers (YNC and CFS). Data extraction will include study information, participant details (sample size, age, gender, AR duration), intervention specifics (acupuncture type, session count, treatment duration), control group data (medication details, acupuncture methods), and outcomes (primary and secondary results, adverse events). Additional information such as main conclusions, funding sources, and acupuncture points will also be documented. Inter-rater reliability, assessing consistency between the two reviewers, will be determined using Cohen's kappa statistics. According to Spector et al., kappa values from 0.0 to 1.0 will categorize consistency from poor to almost perfect at intervals of 0.20, providing a precise measure of agreement. Any discrepancies in data extraction will also be resolved by a third reviewer (XMH). The process will be depicted in a PRISMA flow chart in Figure 1.

### 2.5 Quality assessment

Two independent reviewers (YNC and CFS) will conduct the methodological quality assessment of each randomized controlled trial (RCT) included in this study using the Cochrane Risk of Bias 2 tool (ROB2) (24). Each RCT will be evaluated across five domains: the randomization process, deviations from intended interventions, missing outcome data, accuracy of outcome measurement, and selection of reported results. These domains will be rated as low, unclear, or high risk of bias. To address the handling of high-risk studies, any RCT judged to have “some concerns” in three or more domains, or deemed “high risk” in any domain, will be included in the meta-analysis but will be subject to sensitivity analysis to assess the impact of including these studies. They may also be down-weighted depending on the severity and relevance of the biases identified. This approach allows for a nuanced consideration of studies with potential biases without excluding valuable data outright. In cases of disagreement between the initial reviewers, a third party (XMH) will intervene to mediate and resolve discrepancies. To ensure consistency in quality assessment, this independent assessment will occur at multiple stages of the review process, particularly for subjective ratings like “low” or “high” risk of bias.

In addition to the ROB2 assessment, the strength of the evidence will be evaluated using the Grading of Recommendations Assessment, Development, and Evaluation (GRADE) system (25). Evidence quality will be categorized as high, moderate, low, or very low, based on criteria including risk of bias, inconsistency, indirectness, imprecision, and publication bias. In the event of disagreements during this evaluation, a designated third reviewer (XMH) will intervene to mediate.

### 2.6 Statistical analysis

#### 2.6.1 Pairwise meta-analysis

Pairwise meta-analysis requires a minimum of three studies with identical interventions and outcomes to ensure validity. This analysis will be conducted using RevMan version 5.4 (Review Manager, The Cochrane Collaboration, 2020), calculating effect sizes from baseline changes in symptom scores. Standardized Mean Differences (SMD) for continuous outcomes or Odds Ratios (ORs) for dichotomous outcomes, both with 95% Confidence Intervals (CI), will be used. Heterogeneity will be assessed with Cochran's Q *χ*^2^ test and I2 statistic; values above 50% *I*^2^ or a *p*-value up to 0.10 indicate significant heterogeneity. Sensitivity analyses will exclude studies with excessive heterogeneity to verify result robustness.

#### 2.6.2 Network meta-analysis

Network plots will be generated using Stata V.15.1, which will illustrate the comparative relationships between the different acupuncture interventions. In the network diagram, each node corresponds to a distinct treatment method. The node size correlates with the quantity of studies evaluating that specific intervention. Connections between nodes represent direct comparisons across treatments, whereas the lack of a connection denotes the absence of a direct comparative analysis. This visual representation helps to identify which interventions have been most frequently compared and are thus more robustly represented in the literature.

The NMA will be executed within a Bayesian hierarchical framework using OpenBUGS V.3.2.3 and WinBUGS (version 1.4.3, MRC Biostatistics Unit, Cambridge, UK) to accommodate multiple interventions simultaneously. A random effects model with non-informative priors will be fitted to the data. The comparative efficacy of the interventions will be determined using the Markov chain Monte Carlo (MCMC) method, with three MCMC chains running simultaneously with different initial value sets. After discarding the first 10,000 iterations as burn-in, 60,000 subsequent simulations will be conducted for each chain. Convergence of the models will be assessed visually using the Brooks-Gelman-Rubin diagnostic method, ensuring that the Potential Scale Reduction Factor (PSRF) approaches 1, indicating reliable convergence. The Bayesian framework was selected for its robustness in handling multiple comparisons and sparse data, allowing for the coherent integration of varying types of evidence. This approach not only supports the estimation of model parameters with greater uncertainty but also enhances the interpretability of the treatment efficacy in a probabilistic context.

Treatment effects will be estimated with 95% confidence intervals. The contribution matrix will be employed to display the percentage of direct evidence contributing to each relative effect estimate. The effectiveness of various acupuncture treatments will be ranked using the Surface under the Cumulative Ranking (SUCRA) methodology in Stata V.15.1, which provides a numerical measure of each intervention's relative efficacy. A higher SUCRA score indicates a more effective intervention.

### 2.7 Assessment of inconsistency

When assessing inconsistency in our analysis, we systematically evaluate the consistency between direct and indirect evidence. The node splitting method is currently considered an important tool for this purpose, distinguishing between consistency and inconsistency by comparing the statistical consistency of different data sources. When the *p* value exceeds 0.05, indicating that there is no significant difference between direct and indirect comparisons, a consistent model is applied, confirming the reliability of the network (26). Conversely, the detection of significant inconsistency requires the adoption of an inconsistent model, prompting further investigation of the source of the difference.

### 2.8 Meta-regression, subgroup analysis, and sensitivity analysis

To address study heterogeneity, we will perform meta-regression and subgroup analyses based on predefined variables, such as patient age, treatment duration, and acupuncture technique. A random effects model will also be implemented to accommodate the expected variability across studies, providing a more conservative estimate of the treatment effects. Meta-regression will be utilized to examine how variables such as the initial severity of disease, sample size, average age of participants, and specific aspects of acupuncture treatments—like duration, frequency, and course—affect study outcomes. Subgroup analyses will assess variations across different AR durations to determine if treatment effectiveness varies with the chronicity of the condition. Analyzing different patient age groups will help us understand if age affects the efficacy of acupuncture, potentially guiding age-specific treatment approaches. Furthermore, examining geographical settings (trials conducted in China vs. other countries) will allow us to explore potential cultural or regional differences in acupuncture practice or AR prevalence that might affect treatment outcomes. Multiple sensitivity analyses will be carried out to test the robustness of our findings, evaluating the impact of individual studies and checking the stability of results under various statistical models. Lower-quality studies will be excluded in a repeated meta-analysis to ensure the reliability of our conclusions. This detailed approach will enhance the validity of our findings by precisely accounting for influential factors and differences within study settings.

### 2.9 Assessment of publication biases

Potential publication bias will be carefully assessed for primary outcomes when available from at least 10 studies. This assessment will include the use of funnel plots to visually examine asymmetry, which may suggest bias, and quantitative analysis using Egger's regression test, with a *p*-value less than 0.05 indicating statistically significant publication bias.

We acknowledge the limitations of these methods, especially when fewer than 10 studies are available for a subgroup, which may render funnel plots unreliable. In such cases, we will highlight the potential for increased uncertainty in detecting publication bias and discuss the implications for our findings. When significant publication bias is identified, further analyses will be undertaken to evaluate its impact on the meta-analysis results, ensuring that the final conclusions are both robust and dependable.

### 2.10 Patient and public involvement

This systematic review protocol and NMA uses only publicly available, anonymized data from published clinical studies, involving no direct patient interaction or intervention. However, the formulation of our research questions and determination of outcome measures were informed by preliminary surveys and consultations with patient groups. These engagements helped ensure that our study addresses issues of genuine relevance to patients and the public.

### 2.11 Ethics and dissemination

Ethical approval is not required for this secondary data analysis. The results will be peer-reviewed and intended for publication.

## 3 Discussion

AR is an inflammatory disorder of the nasal mucosa mediated by IgE (27). The prevalence of AR has been increasing annually, significantly impacting people's lives. The pathophysiology and treatment mechanisms of AR have been extensively studied, yet many aspects remain unclear and require further investigation (28). Treatment for AR is divided into pharmacological and non-pharmacological approaches. Among non-pharmacological treatments, acupuncture has been shown by a substantial body of clinical research to be effective in treating AR (26). Evidence suggests that the efficacy of acupuncture in treating AR may be related to its regulation of autonomic nerve functions, neuroendocrine, and immune networks (20, 29, 30). However, high-quality evidence-based medical evidence for the optimal acupuncture treatment protocol for AR is lacking.

NMA enables the simultaneous evaluation of multiple treatment options by integrating both direct and indirect comparisons across varied studies (31). In this protocol, we restrict inclusion to studies published in either English or Chinese. This criterion may limit the diversity of the data and introduce a potential selection bias. Our NMA will clearly define the types of acupuncture therapies included and their combined use, aiming to comprehensively evaluate their efficacy in managing AR, thereby providing more robust evidence (32). To our knowledge, this research represents the initial application of NMA to evaluate acupuncture treatments for AR. Despite this limitation, our proposed approach will offer a comparative analysis of different acupuncture techniques, providing clinical evidence to support evidence-based medical decisions in the treatment of AR. By systematically assessing and comparing the effectiveness of these acupuncture techniques, our study will help clinicians tailor treatments to individual patient needs, potentially improving patient outcomes and satisfaction. Furthermore, our findings will inform future guidelines and policy-making by identifying which acupuncture approaches are most effective. This enhanced approach will help identify the most effective acupuncture protocols, ensuring that the findings are clinically relevant and applicable in practice.

This NMA has several limitations. The exclusive inclusion of studies published in English or Chinese might introduce a language bias, thereby limiting the generalizability of our results. Moreover, inherent flaws in the original studies, such as potential biases arising from non-blinded assessments and the presence of small sample sizes, could undermine the robustness of our conclusions. Furthermore, the variability in acupuncture techniques and the expertise of practitioners across studies may introduce substantial heterogeneity, complicating the interpretation and application of the findings.
